# Establishment of Murine Pregnancy Requires the Promyelocytic Leukemia Zinc Finger Transcription Factor

**DOI:** 10.3390/ijms25063451

**Published:** 2024-03-19

**Authors:** Lan Hai, Vineet K. Maurya, Francesco J. DeMayo, John P. Lydon

**Affiliations:** 1 Department of Molecular and Cellular Biology, Baylor College of Medicine, One Baylor Plaza, Houston, TX 77030, USA; lanhai@hsph.harvard.edu (L.H.); vineet.maurya@bcm.edu (V.K.M.); 2 Reproductive and Developmental Biology Laboratory, National Institute of Environmental Health Sciences, Research Triangle Park, Durham, NC 27709, USA; francesco.demayo@nih.gov

**Keywords:** promyelocytic leukemia zinc finger, mouse, female infertility, implantation, decidualization, progesterone

## Abstract

Using an established human primary cell culture model, we previously demonstrated that the promyelocytic leukemia zinc finger (PLZF) transcription factor is a direct target of the progesterone receptor (PGR) and is essential for progestin-dependent decidualization of human endometrial stromal cells (HESCs). These in vitro findings were supported by immunohistochemical analysis of human endometrial tissue biopsies, which showed that the strongest immunoreactivity for endometrial PLZF is detected during the progesterone (P4)-dominant secretory phase of the menstrual cycle. While these human studies provided critical clinical support for the important role of PLZF in P4-dependent HESC decidualization, functional validation in vivo was not possible due to the absence of suitable animal models. To address this deficiency, we recently generated a conditional knockout mouse model in which PLZF is ablated in PGR-positive cells of the mouse (*Plzf ^d/d^*). The *Plzf ^d/d^* female was phenotypically analyzed using immunoblotting, real-time PCR, and immunohistochemistry. Reproductive function was tested using the timed natural pregnancy model as well as the artificial decidual response assay. Even though ovarian activity is not affected, female *Plzf ^d/d^* mice exhibit an infertility phenotype due to an inability of the embryo to implant into the *Plzf ^d/d^* endometrium. Initial cellular and molecular phenotyping investigations reveal that the *Plzf ^d/d^* endometrium is unable to develop a transient receptive state, which is reflected at the molecular level by a blunted response to P4 exposure with a concomitant unopposed response to 17-β estradiol. In addition to a defect in P4-dependent receptivity, the *Plzf ^d/d^* endometrium fails to undergo decidualization in response to an artificial decidual stimulus, providing the in vivo validation for our earlier HESC culture findings. Collectively, our new *Plzf ^d/d^* mouse model underscores the physiological importance of the PLZF transcription factor not only in endometrial stromal cell decidualization but also uterine receptivity, two uterine cellular processes that are indispensable for the establishment of pregnancy.

## 1. Introduction

Although embryo abnormalities are a major cause of preclinical pregnancy loss [1,2,3,4,5], an increasing number of studies implicate the endometrium as an additional contributing factor in embryo implantation failure and early embryo miscarriage, reviewed in [6,7,8,9,10,11,12]. Given that assisted reproductive technologies (ARTs) depend on the transfer of healthy embryos into a receptive endometrium, a non-receptive endometrium at the time of embryo transfer is thought to also undercut the full potential of ART-conceived pregnancies [13,14,15,16,17]. In addition, a non-receptive endometrium is implicated as one of a number of factors leading to recurrent pregnancy loss [18,19], defined in the United States as two or more consecutive pregnancy losses that are diagnosed by ultrasound and/or histopathology [20]. Termed the adverse ripple effect [21,22], incomplete progression of the normal cellular and molecular changes in the endometrium during the peri-implantation period has also been linked with initiating adverse outcomes that symptomatically manifest in the subsequent trimesters of pregnancy; these include pre-eclampsia, placental insufficiency, intrauterine fetal restriction, and preterm birth [21].

From primate to rodent, the process of embryo implantation sequentially progresses through defined interdependent developmental stages, ostensibly beginning with blastocyst apposition and attachment to the luminal epithelium of the receptive endometrium, then embryo invasion into the subepithelial stroma, followed by decidualization of a surrounding zone of stromal fibroblasts into specialized epithelioid decidual cells; reviewed in [21,23]. Surrounding the conceptus, decidual cells support embryonic development and invasion as well as furnish protection against an adverse cytotoxic microenvironment until placentation [9].

Progesterone (P4), through the progesterone receptor (PGR), is indispensable for the majority of these early endometrial cellular changes that are required for embryo implantation, stromal decidualization, and subsequent development of the maternofetal interface [24]. Although our understanding of endometrial P4 responsiveness at the cellular level is significantly advanced, the molecular underpinnings of these cellular responses remain incomplete. A member of the evolutionary conserved POK (POZ and Kruppel) family of C_2_H_2_-type zinc finger transcription factors [25,26], the promyelocytic leukemia zinc finger (PLZF; also known as ZBTB16 or ZNF 145) can act as a transcriptional activator or repressor depending on cell and signaling context [26,27,28,29,30,31,32,33,34,35]. The PLZF transcription factor mediates a wide spectrum of early developmental programs as well as physiological effects in the adult, ranging from skeletal patterning, innate immune cell development, and hematopoiesis to spermatogenesis, reviewed in [26]. Supporting these various physiologies, PLZF controls the expansion of the stem cell pool and its differentiation, regulates cellular crosstalk, as well as governs cell proliferation, differentiation and programmed cell death, reviewed in [26].

Through an integrative approach using ChIP-seq (chromatin immunoprecipitation followed by deep sequencing) and RNA-seq datasets derived from human endometrial stromal cells (HESCs), PLZF was previously identified as a direct target of the PGR [33]. Subsequent cell-based studies demonstrated that progestin-induction of PLZF not only occurs in HESCs but was required for progestin-dependent HESC decidualization in cell culture [33,35]. This clinically significant in vitro result, along with immunohistological data showing PLZF expression occurs in human endometrial tissue, specifically during the P4-dominant secretory phase of the menstrual cycle [33], indicated that PLZF exerts an important P4 mediator role in the mammalian endometrium in vivo.

To test this proposal in vivo, a new bigenic mouse model (*Plzf ^d/d^*) was generated in which PLZF function is conditionally ablated in PGR-positive cells by crossing our previously reported *Pgr ^cre^* knockin mouse [36] with our recently generated *Plzf* floxed (*Plzf ^f/f^*) mouse in which exon 2 of the *Plzf* gene is floxed [37]. Comparative histomorphometric, immunohistochemical, cellular, and molecular studies demonstrate that PLZF is essential for P4-dependent endometrial stromal decidualization in vivo. Furthermore, our studies on the *Plzf ^d/d^* bigenic strongly support an important role for PLZF in P4-dependent endometrial receptivity, which is essential for embryo implantation and the early establishment of the maternofetal interface. Together, our mouse studies described here, along with our previously published HESC investigations [33,35], strongly support the hypothesis that the evolutionally conserved endometrial PLZF transcription factor is critical in mediating both P4-dependent uterine receptivity and decidualization.

## 2. Results

### 2.1. The PLZF Transcription Factor Is Significantly Expressed in the Murine Decidual Cell Population

**Figure 1:**
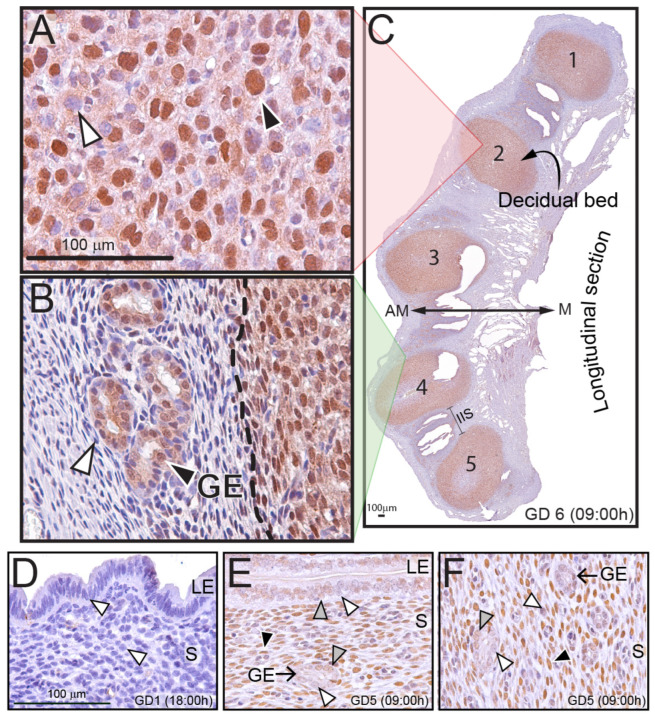
The murine decidua expresses the PLZF transcription factor. (**A**) Immunohistochemistry detects PLZF expression in the majority of decidual cells in the decidual bed of the mouse at GD 6 (black arrowhead); white arrowhead indicates a small subset of decidual cells that are PLZF negative. (**B**) Immunohistochemistry also reveals PLZF expression in the majority of glandular epithelial (GE) cells (black arrowhead), whereas the majority of stromal fibroblasts in the inter-implantation site (IIS) region express low levels of PLZF (white arrowhead); the vertical dashed line indicates the border between the periphery of the decidua and the IIS region. The scale bar in (**A**) applies to (**B**). (**C**) A longitudinal section of the uterine horn with five decidual beds (numbered 1–5), which is immunohistochemically stained for PLZF expression, is shown. The image in (**C**) was captured at low magnification of one uterine horn from which the higher magnification immunohistochemical data shown in panels (**A**,**B**) are derived. The antimesometrial and mesometrial poles are indicated by AM and M, respectively; one of the IIS regions is indicated by a bracket. (**D**) There is an absence of obvious PLZF immunopositivity in the murine uterus on the afternoon of GD 1 (white arrowheads); LE and S denote luminal epithelium and stroma, respectively. (**E**) On the morning of GD5, low immunopositivity for PLZF can be detected in a subset of luminal epithelial cells (grey arrowhead), while the remaining luminal epithelial cell population scores negative for PLZF expression (white arrowhead). The glandular epithelium (GE) exhibits a similar PLZF pattern as described for the luminal epithelium. The black arrowhead indicates a PLZF positive stromal cell. (**F**) The majority of the cells in the stromal (S) compartment of the GD5 endometrium are strongly immunopositive for PLZF expression (black arrowhead), whereas only a small subset of stromal cells is PLZF negative (white arrowhead). The grey arrowhead indicates a glandular epithelial cell that is positive for PLZF expression. The scale bar in (**D**) applies to both panels (**E**,**F**).

To determine the expression pattern of PLZF in the endometrium of the pregnant mouse, immunohistochemical analysis was performed (Figure 1A–C). On the morning of gestation day 6 (GD6), the majority of decidual cells are immunopositive for PLZF expression within the murine decidua (Figure 1A). Immunopositivity for PLZF is located exclusively in the nucleus of decidual cells (Figure 1A), which follows a similar cellular spatial staining pattern in HESCs [33]. Outside the decidua, endometrial stromal cells within the inter-implantation sites (IIS) express significantly lower levels of PLZF, whereas a subset of glandular epithelial cells display strong PLZF expression (Figure 1B). Similar to the virgin uterus [33], PLZF expression is not detected in the murine endometrium at GD1 (Figure 1D), whereas PLZF expression is clearly evident in pre-decidual stromal fibroblasts at GD5 (Figure 1E,F). Interestingly, lower levels of PLZF are also detected in a subset of luminal and glandular epithelial cells within this tissue (Figure 1E,F). Immunohistochemical data shown in Supplementary Figure S1A–E further confirms that PLZF is expressed not only in fully differentiated decidual cells at GD6 in the mouse but also in pre-decidual stromal fibroblasts and at low levels in the luminal and glandular epithelial cellular compartments at GD5. The specificity of the PLZF antibody was confirmed using a positive control (testicular tissue derived from two-week-old male mice [38]), Supplementary Figure S1F,G.

### 2.2. The Plzf ^d/d^ Bigenic Uterus Is Significantly Unresponsive to Progesterone

**Figure 2:**
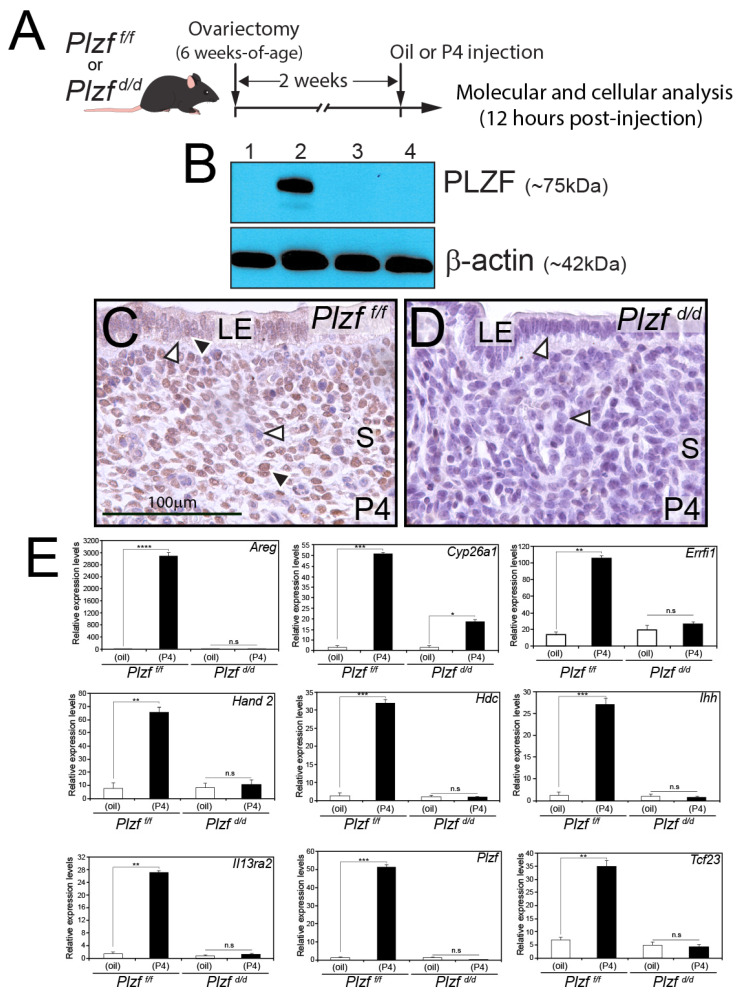
The PLZF transcription factor is not induced in the *Plzf ^d/d^* uterus in response to acute progesterone exposure. (**A**) The schematic shows the P4 hormone treatment regimen for ovariectomized *Plzf ^f/f^* control and *Plzf ^d/d^* mutant mice that were used to induce uterine PLZF protein expression. Two weeks following ovariectomy, 8-week-old control and mutant mice received either P4 (1 mg) or hormone vehicle (sesame oil) through intrascapular s.c. injection. Mice were euthanized 12 h post-injection before uterine tissues were removed and processed for cellular and molecular analysis. (**B**) Shown are the PLZF immunoblot results for *Plzf ^f/f^* mice treated with oil (lane 1), *Plzf ^f/f^* mice treated with P4 (lane 2), *Plzf ^d/d^* mice treated with oil (lane 3), and *Plzf ^d/d^* mice treated with P4 (lane 4). Note: For each gel lane, uterine tissue protein was pooled from four mice per genotype and treatment group; β-actin served as a loading control. As expected, P4-induction of PLZF protein (~75 kDa) was detected only in the P4-treated *Plzf ^f/f^* mouse group (lane 2). The absence of an immunoreactivity band for PLZF in the uterine protein preparation from the P4-treated *Plzf ^d/d^* mouse group (lane 4) confirms that the engineered *Plzf ^d/d^* mouse operates as designed. (**C**) Supporting the immunoblot data in panel (**B**), immunohistochemistry clearly detects PLZF protein expression in a subset of endometrial stromal (S) fibroblasts and luminal epithelial (LE) cells (black arrowhead) in the P4-treated *Plzf ^f/f^* mouse (black arrowhead); stromal fibroblasts and epithelial cells that are PLZF negative are indicated by white arrowheads. (**D**) Confirming the immunoblot results in panel (**B**), endometrial PLZF protein is not detected by immunohistochemistry in the P4 treated *Plzf ^d/d^* mouse (white arrowheads); the scale bar in (**C**) applies to panel (**D**). (**E**) Histograms display the results from the qRT-PCR analysis of expression from the following P4-responsive genes: Amphiregulin (*Areg*) [40]; Cytochrome P450 26A1 (*Cyp26a1*) [41]; ERBB receptor feedback inhibitor 1 (*Errfi1*) [42]; Heart and neural crest derivatives expressed 2 (*Hand 2*) [43]; Histidine decarboxylase (*Hdc*) [44]; Indian hedgehog (*Ihh*) [45]; Interleukin-13 receptor subunit alpha-2 (*Il13ra2*) [46]; promyelocytic leukemia zinc finger (*Plzf*) [33]; and Transcription factor 23 (*Tcf 23*) [47] in *Plzf ^f/f^* and *Plzf ^d/d^* uterine tissue following the P4 treatment described in panel (**A**). Note that for many of these P4-responsive genes, induction by P4 is either significantly attenuated or blocked in the *Plzf ^d/d^* uterus (*n* = 4 mice per genotype and treatment). Not significant is denoted by n.s. whereas *, **, *** and **** indicate *p* values of <0.05, <0.01, <0.001 and <0.0001 respectively.

To determine PLZF’s role in murine endometrial biology, a new bigenic mouse model was generated in which PLZF is specifically ablated in cells that express the PGR. By crossing our previously reported *Pgr ^cre^* mouse [36] with a new mouse model in which exon 2 of the *Plzf* gene is floxed (*Plzf ^f/f^*) [37], the early skeletal developmental abnormalities of the *Plzf ^KO^* mouse can be avoided in the resultant conditional bigenic, termed the *Plzf ^d/d^* mouse hereon. Encoding greater than 50% of the PLZF protein [39] that encompasses the initiating ATG, the N-terminal BTB/POZ domain, and the first two C_2_H_2_ zinc fingers of the PLZF DNA binding domain, the exon 2 deletion effectively abrogates PLZF expression in the *Plzf ^KO^* mouse [39] and in a mouse model in which *Plzf ^f/f^* is crossed with the global *cytomegalovirus* promoter-driven cre (*CMV ^cre^*) mouse [37]. To confirm that PLZF is not expressed in the uterus of the P4-treated *Plzf ^d/d^* mouse, an established P4 treatment regimen on ovariectomized mice was used (Figure 2A) [33,37].

Western immunoblot analysis clearly shows that the PLZF protein is not induced in the *Plzf ^d/d^* uterus in response to acute P4 exposure (Figure 2B), a result which is also confirmed by immunohistochemistry (Figure 2C,D). While the P4-treated *Plzf ^f/f^* uterus shows PLZF expression in endometrial stromal cells with low-level PLZF expression in a subset of luminal epithelial cells (Figure 2C), the *Plzf ^d/d^* endometrium is devoid of PLZF immunoreactivity (Figure 2D). Because PLZF is not expressed in the P4-treated *Plzf ^d/d^* endometrium (Figure 2B,D), we asked whether the expression of other P4 target genes is compromised in the absence of PLZF. Using quantitative real-time PCR (qRT-PCR), we demonstrate that the expression of the majority of the P4 responsive genes tested (Figure 2E) is either not induced or minimally induced by P4 in the *Plzf ^d/d^* endometrium (Figure 2E). Together, these results confirm that the *Plzf ^d/d^* mouse operates as designed and that the artificial induction of a significant number of P4 responsive genes in the ovariectomized mouse model is compromised in the *Plzf ^d/d^* endometrium.

### 2.3. The Plzf ^d/d^ Bigenic Endometrium Fails to Support Embryo Implantation

To determine whether endometrial PLZF has a functional role during the early stages of pregnancy establishment, a standard breeding trial for at least a 6-month period was conducted using age-matched *Plzf ^f/f^* and *Plzf ^d/d^* females housed with fertility-proven stud males. The results clearly show that while *Plzf ^f/f^* mice produce the expected number of litters with normal numbers of pups, the *Plzf ^d/d^* female does not produce pups during this time period (Table 1).

The infertility phenotype displayed by the *Plzf ^d/d^* female mouse occurs despite exhibiting normal ovarian activity, producing pre-implantation blastocysts similar in number to the *Plzf ^f/f^* mouse, and having P4 and estradiol (E2) serum levels that are equivalent to levels detected in the *Plzf ^f/f^* mouse at GD 5 (Supplementary Figure S2).

**Figure 3:**
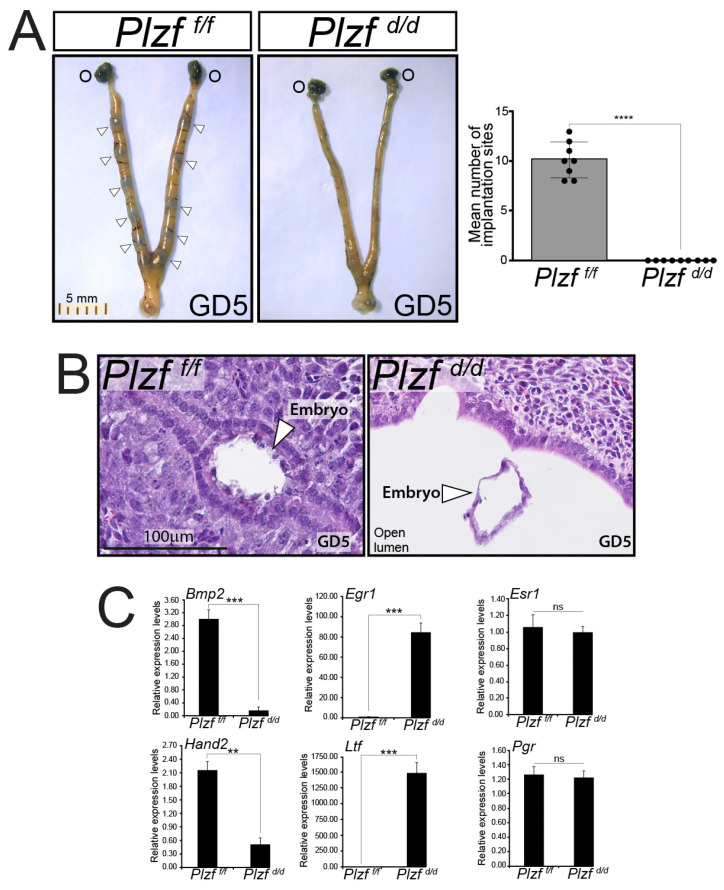
Embryo implantation is impaired in the *Plzf ^d/d^* mouse. (**A**) The left panel shows the gross morphology of the uterus obtained from the *Plzf ^f/f^* control mouse at GD5. The *Plzf ^f/f^* mouse received a tail vein injection of a Chicago Sky Blue dye solution minutes prior to euthanasia and tissue harvesting. Note the locations of implantation sites along each uterine horn as indicated by the intense Chicago Sky Blue staining (white arrowheads); the position of the stained ovaries is indicated by O. The right panel shows the gross morphology of the uterus derived from the similarly hormone treated *Plzf ^d/d^* mutant at GD5. Note the clear absence of implantation sites along both *Plzf ^d/d^* uterine horns, with only the ovaries scoring positive for Chicago Sky Blue staining. The histogram on the far right quantitatively displays the mean number of implantation sites per mouse in the *Plzf ^f/f^* control group (*n* = 8); no implantation sites were observed in the *Plzf ^d/d^* mutant group (*n* = 9). (**B**) The left panel shows a transverse uterine tissue section with an embryo from a *Plzf ^f/f^* mouse at GD5; the tissue section is stained with H&E. Note the typical tight attachment of the embryo to the apical surface of the epithelial lining of the *Plzf ^f/f^* endometrium (white arrowhead). The right panel shows a representative tissue section of a *Plzf ^d/d^* uterus with an embryo at GD5, which is also stained with H&E. Note the absence of embryo implantation, but instead, the embryo floats in the open lumen (white arrowhead). (**C**) Quantitative real-time PCR analysis reveals that the expression of genes associated with murine uterine receptivity and P4-responsiveness (i.e., Bone morphogenetic protein 2 (*Bmp 2* [49,50]) and *Hand 2* [43]) is significantly attenuated in the *Plzf ^d/d^* uterus when compared with the *Plzf ^f/f^* uterus at GD5. Conversely, E2-responsive uterine biomarkers (i.e., Lactoferrin (*Ltf*) [51] and Early growth response 1 (*Egr1*) [33,52,53,54,55]) are significantly elevated in the *Plzf ^d/d^* uterus when compared with the *Plzf ^f/f^* uterus at GD5. Importantly, the levels of Estrogen receptor 1 (*Esr1*) and *Pgr* expression are not significantly altered between the *Plzf ^f/f^* and *Plzf ^d/d^* uteri at GD5. Not significant is denoted by ns whereas **, ***, and **** indicate *p* values of <0.01, <0.001, <0.0001 respectively.

Using a timed natural pregnancy model [48], implantation sites are clearly visible following tail vein injection of Chicago Sky Blue stain along both uterine horns of the *Plzf ^f/f^* mouse at GD5 (Figure 3A). In contrast, implantation sites are not detected in the *Plzf ^d/d^* uterus at GD5 using the same staining procedure (Figure 3A). Follow-up immunohistochemical approaches show that while the embryo attaches to the apical surface of the luminal epithelium of the *Plzf ^f/f^* endometrium at GD 5 (Figure 3B), embryo attachment does not occur in the *Plzf ^d/d^* endometrium but instead floats in the open lumen (Figure 3B). Interestingly, the expression levels of a number of P4-responsive genes (Bone morphogenetic protein 2 (*Bmp2*) [49] and Heart and neural crest derivatives expressed 2 (*Hand2*) [43]), which are linked with the endometrium’s ability to support embryo implantation, are significantly attenuated in the *Plzf ^d/d^* endometrium as compared with the *Plzf ^f/f^* endometrium at GD 5 (Figure 3C).

Conversely, genes associated with E2-signaling (i.e., lactoferrin (*Ltf*) [51] and early growth response 1 (*Egr1*) [33,52,53,54,55]) in the endometrium are markedly elevated in the *Plzf ^d/d^* endometrium as compared with the *Plzf ^f/f^* endometrium (Figure 3C). These perturbations in the expression of P4 and E2 responsive genes in the *Plzf ^d/d^* endometrium occur in the absence of significant changes in the expression of the estrogen receptor-α (*Esr 1*) and *Pgr* (Figure 3C). Collectively, these data support a critical role for endometrial PLZF in the early stages of embryo implantation and that the absence of PLZF derails normal E2 and P4 responsiveness of the endometrium at the molecular level.

### 2.4. Endometrial Receptivity Requires PLZF

**Figure 4:**
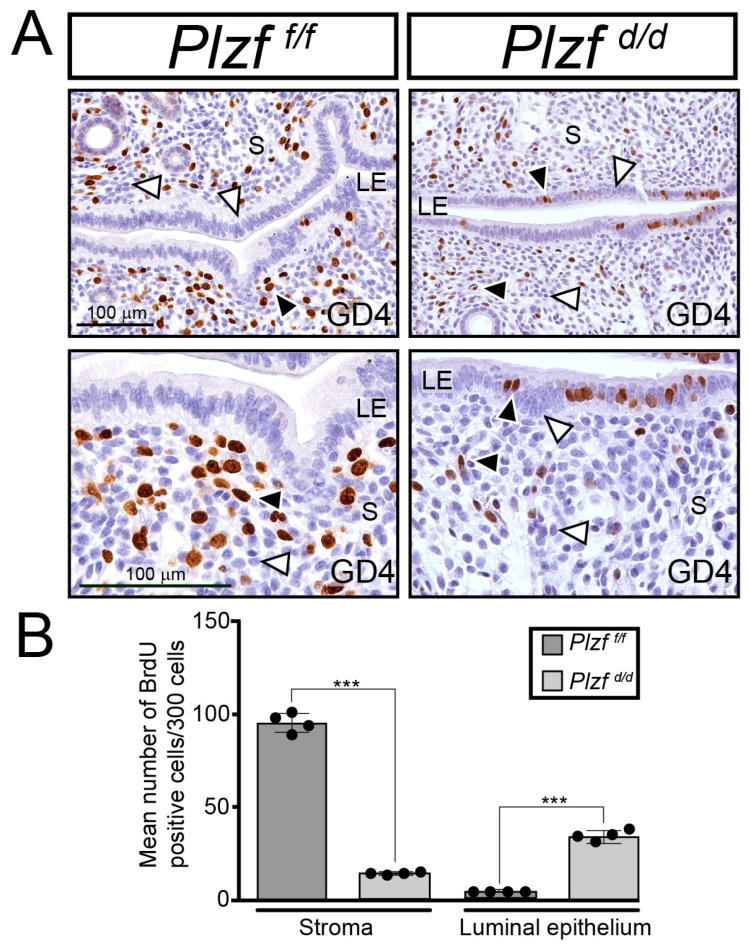
Uterine receptivity is impaired in the *Plzf ^d/d^* mouse at GD4. (**A**) The left panels display the distribution of cells that are immunohistochemically positive for BrdU incorporation in the endometrium of the *Plzf ^f/f^* mouse at GD4. The bottom panel is a higher magnification of a region shown in the top panel. Note that the majority of cells are BrdU negative in the luminal epithelium (LE), whereas a subset of stromal (S) cells is BrdU positive (black arrowhead), another subset of stromal cells is BrdU negative (white arrowhead). The right panels show the distribution of cells positive for BrdU incorporation in the endometrium of the *Plzf ^d/d^* mouse at GD4. Again, the bottom panel shows a higher magnification of a region shown in the top panel. Note the retention of a significant number of BrdU-positive cells in the *Plzf ^d/d^* luminal epithelium (black arrowhead). In addition, there is a marked decrease in the number of BrdU-positive stromal cells in the *Plzf ^d/d^* endometrium (black arrowhead) at GD4; white arrowheads denote cells that are negative for BrdU. Note: scale bars in the left top and bottom panels also apply to the right top and bottom panels, respectively. (**B**) The histogram displays the quantitation of the relative mean number of BrdU-positive cells in the stromal and luminal epithelial compartments of the *Plzf ^f/f^* and *Plzf ^d/d^* uteri at GD4 (*n* = 4/genotype/cellular compartment); *** indicate a *p* value of <0.001.

Because of the impaired responsiveness of the *Plzf ^d/d^* endometrium to both P4 and E2 (Figure 3C) and because sequential E2 and P4 exposure of the murine endometrium is critical for the development of the transient receptive state at GD4 [21], the cellular proliferative status of the *Plzf ^f/f^* and *Plzf ^d/d^* endometrium at GD4 was examined (Figure 4).

At GD4, pre-ovulatory E2-induced epithelial proliferation is significantly suppressed by rising levels of post-ovulatory P4 hormone in the normal murine endometrium [21]. As expected at GD4, the endometrium of the *Plzf ^f/f^* control mouse displays a low number of epithelial cells that score positive for BrdU incorporation (Figure 4A (left panels)), whereas numerous fibroblastic cells are BrdU positive in the stromal compartment. In contrast, the *Plzf ^d/d^* endometrium still retains a subset of epithelial cells that score BrdU positive as well as displaying a stromal compartment that is less proliferative than the *Plzf ^f/f^* stroma at this time of gestation (Figure 4A (right panels)). These results were also confirmed using an established E2 and P4 hormone treatment protocol on ovariectomized mice that elicits an artificial receptive state [56], albeit underpinned by an exaggerated cellular proliferative response (Supplementary Figure S3). Taken together, these results reveal an unexpected role for pre-decidual PLZF in the development of the receptive endometrium that occurs prior to the development of the endometrial decidua.

### 2.5. Decidualization of the Murine Endometrium Is Dependent on PLZF

**Figure 5:**
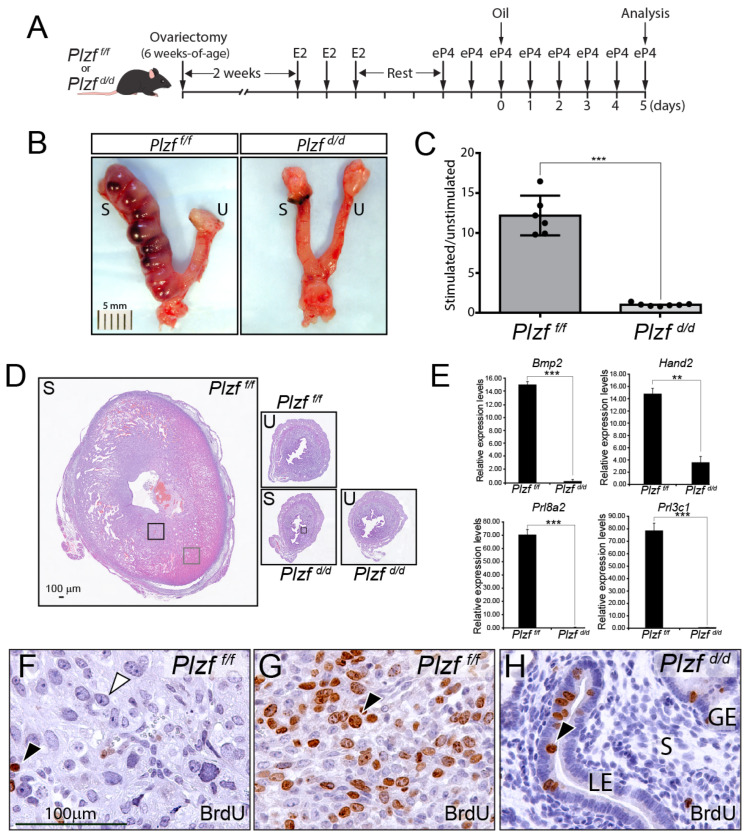
Decidualization of the mouse uterus requires the PLZF transcription factor. (**A**) The schematic depicts the standard hormone treatment of ovariectomized *Plzf ^f/f^* and *Plzf ^d/d^* mice to prepare uterine tissue for an artificial decidual response following instillation of sesame oil (the deciduogenic stimulator) in one uterine horn. (**B**) The left panel shows the gross morphology of the *Plzf ^f/f^* uterus following the hormone treatment regimen shown in panel (**A**). Note the appearance of the typical deciduomata in the stimulated (S) uterine horn, whereas the contralateral unstimulated (U) uterine horn does not display this tissue response. The right panel shows the similarly treated *Plzf ^d/d^* uterus. Note the clear absence of a decidual response in the *Plzf ^d/d^* uterine horn (S) that received the deciduogenic stimulus. (**C**) The histogram displays the means for the wet weight ratio of stimulated/unstimulated for *Plzf ^f/f^* (*n* = 6) and *Plzf ^d/d^* (*n* = 7) uteri in this study. (**D**) The left panel shows the transverse tissue section of the *Plzf ^f/f^* stimulated (S) horn that is stained with H&E. The top right panel shows the corresponding unstimulated (U) uterine horn from the same *Plzf ^f/f^* mouse. The bottom right panels show the stimulated (S) and unstimulated (U) uterine horns from the *Plzf ^d/d^* mouse. Note that the size between the stimulated and unstimulated *Plzf ^d/d^* horns is indistinguishable. The scale bar in the left panel applies to all panels in this panel series. (**E**) Quantitative real-time PCR analysis shows that typical gene expression levels associated with the uterine decidual response (i.e., *Bmp2* [49,50], *Hand2* [43], Prolactin family 3, subfamily c, member 1 (*Prl3c1*) [61] and Prolactin family 8, subfamily a, member 2 (*Prl8a2*) [62]) are significantly decreased in the *Plzf ^d/d^* stimulated uterine horn when compared with the *Plzf ^f/f^* stimulated horn; ** and *** indicate *p* values of <0.01 and <0.001 respectively (**F**) The tissue section of the *Plzf ^f/f^* stimulated horn is stained for BrdU incorporation; the tissue section is located in the black square shown in panel (**D**) left panel. Note the presence of large decidual cells (white arrowhead), with only a few cells scoring positive for BrdU immunoreactivity (black arrowhead). (**G**) The tissue section stained for BrdU incorporation from the *Plzf ^f/f^* stimulated uterine in a region indicated by the grey box in panel (**D**) left panel. Note the numerous cells positive for BrdU staining in the expanding cellular front of the deciduomata (black arrowhead). (**H**) The tissue section in the *Plzf ^d/d^* stimulated uterine horn in a region indicated by the black box in panel (**D**) (bottom left panel); note that the majority of stromal cells are negative for BrdU incorporation, whereas the epithelial compartment still retains cells positive for BrdU (black arrowhead); LE, GE, and S denote luminal epithelium, glandular epithelium, and stroma, respectively. The scale bar in panel (**F**) also applies to panels (**G**,**H**).

Because the endometrial phenotype of the *Plzf ^d/d^* mouse manifests during the receptive period prior to full decidualization of the stroma, the artificial decidual response assay had to be used (Figure 5A). Using an established E2 and P4 hormone treatment regimen on ovariectomized mice that prepares the uterine horn to undergo a decidual response [57], the *Plzf ^d/d^* uterus is shown to be incapable of launching a decidual response, unlike the uterus of a similarly treated *Plzf ^f/f^* mouse (Figure 5A–D). At the gross morphological level, the *Plzf ^d/d^* uterus fails to generate the typical deciduomata in response to the artificial deciduogenic stimulus (Figure 5A–D), in this case, sesame oil instillation into one uterine horn. The absence of a decidual response in the *Plzf ^d/d^* endometrium is also accompanied by a significant decrease in the levels of established markers of murine decidualization, such as *Bmp2*, *Hand2*, *Prl8a2*, and *Prl3c1* (Figure 5E) [43,49,50,58,59,60,61,62]. At the cellular level, there is a conspicuous absence of large epithelioid stromal decidual cells in the oil-treated *Plzf ^d/d^* uterine horn, but instead, the retention of proliferative cells in the epithelium is clear (Figure 5F,H). Apart from furnishing long sought-after in vivo support for our previous human cell-based findings showing a critical role for PLZF in HESC decidualization [33,35], these results—in combination with our findings reported in Figure 4—provide strong support for PLZF’s importance not only in endometrial decidualization but also endometrial receptivity.

## 3. Discussion

Depending on tissue type and signaling context, PLZF has been shown to be rapidly induced by the following steroid hormones: aldosterone, cortisone, glucocorticoid, and testosterone [32,63,64,65]. These findings are significant because, like P4, these steroids exert their physiological effects through their closely related nuclear receptors of the subfamily 3 (group C) of the nuclear receptor superfamily [66]. A recent retrospective analysis of past microarray datasets revealed that support already existed as early as 2002 for the rapid induction of *Plzf* transcripts by P4 in the uterus of the ovariectomized mouse (Gene Expression Omnibus (GEO) profiles: *Zbtb16*-Progesterone effects on uterus time course (ncbi.nlm.nih.gov/geoprofiles/913691) accessed on 12 July 2023). Moreover, *PLZF* induction during the P4-dominant early- to mid-secretory phase of the human menstrual cycle was also previously documented in a separate microarray study (GEO profiles: *ZBTB16*-Endometrium throughout the menstrual cycle (ncbi.nlm.nih.gov/geoprofiles/?term=24460831) accessed on 12 July 2023). Together, these early microarray datasets provided the first indication that PLZF is an early molecular target of P4 signaling in the murine uterus and that the levels of endometrial PLZF expression are significantly elevated during the P4-dependent secretory phase of the menstrual cycle, raising the question of whether P4 induction of endometrial PLZF is essential for early pregnancy establishment. More recently, our group provided in vitro functional support for this proposal using a primary HESC culture model [33], which demonstrated that rapid progestin-induction of PLZF from basal levels in pre-decidual HESCs is essential for progestin-dependent HESC decidualization. Our subsequent in vitro studies also revealed that basal levels of PLZF in pre-decidual HESCs are essential for their proliferation, migration, and invasion [35], cellular properties known to be displayed by HESCs as they decidualize [67].

Although studies on cultured primary HESCs provided essential cellular and molecular insights into the role of PLZF in HESC decidualization, whether these in vitro functional findings translated to the in vivo situation remained an open question. Because the *Plzf ^KO^* mouse exhibits a severe skeletal patterning defect [39,68], which is also observed in humans [69], we took advantage of our recently generated conditional *Plzf ^d/d^* mouse that circumvents these confounding phenotypes [37]. While the initial objective in using this mouse model was to confirm our in vitro findings that PLZF is crucial for endometrial stromal cell decidualization, our first in vivo results here show that PLZF expression in pre-decidual stromal fibroblasts is critical for the development of endometrial receptivity for embryo implantation, a developmental step that precedes decidualization. Our initial molecular analysis reveals that the induction of the majority of P4-responsive molecular targets tested in this study is significantly attenuated when PLZF is absent during the peri-implantation period. Therefore, this molecular result further supports PLZF as an apex P4 mediator that is positioned high in the hierarchy of immediate P4-responsive target genes. At the cellular level, the attenuated P4 responsiveness of the *Plzf ^d/d^* endometrium manifests as an incomplete suppression of E2-induced epithelial proliferation; such an unchecked heightened E2 response has been linked to implantation failure in a number of mouse models [70,71,72] and implicated in human implantation failure [73,74,75,76]. Moreover, the significant decrease in the number of proliferating pre-decidual stromal cells in the *Plzf ^d/d^* endometrium at this time parallels similar findings from our previous HESC studies [33,35], where PLZF is required for P4-dependent expansion of the pre-decidual stromal cell population prior to its decidualization. The above aberrant cellular responses are underscored at the molecular level by a marked increase in the levels of E2-dependent molecular targets in *Plzf ^d/d^* endometrium. In particular, the detection of elevated *Egr1* levels in the *Plzf ^d/d^* uterus is interesting as P4-induction of PLZF has been shown to directly suppress *Egr1* expression during HESC decidualization [33]. Our past studies also demonstrate that while EGR1 expression in pre-decidual stromal fibroblasts is essential for these cells to later decidualize [35], the suppression of EGR1 expression by P4-induction of PLZF is a prerequisite if these pre-decidual stromal fibroblasts are to develop into decidual cells. As reported for HESCs [33], the levels of ESR1 and PGR are not altered in the *Plzf ^d/d^* endometrium to account for these molecular changes.

Because of our past human studies [33,35], our initial objective in using the *Plzf ^d/d^* mouse was to determine the in vivo importance of PLZF in murine endometrial stromal cell decidualization, which normally occurs at GD6 onwards [77]. To bypass the earlier *Plzf ^d/d^* endometrial receptivity phenotype, the well-described murine artificial decidual response assay was used to determine PLZF’s endometrial intrinsic role in the decidualization of this tissue [48]. Supporting our previous human cell culture studies [33,35], the artificial decidual response assay confirmed a critical in vivo role for endometrial PLZF in the terminal differentiation of endometrial stromal fibroblasts into decidual cells. Taken together, these *Plzf ^d/d^* mouse findings here underscore at least two important roles for PLZF during the peri-implantation period: (i) development of uterine receptivity to allow embryo attachment to the apical surface of the luminal epithelium and (ii) endometrial stromal fibroblast decidualization, occurring a day later in the pregnant mouse [21].

Apart from PLZF expression in pre-decidual and terminally differentiated decidual stromal cells of the murine uterus during early pregnancy, our immunohistochemical data reveal that PLZF is expressed in a subset of cells both in the luminal and glandular epithelial compartments. These observational findings indicate that PLZF may exert a previously unsuspected uterine cell-type specific role in the establishment of the maternofetal interface. With the availability of the *Plzf ^f/f^* model [37], addressing the cell-type specific involvement of PLZF in uterine receptivity and decidualization is now feasible due to the recent generation of uterine cell-type specific cre mouse models. Indeed, these cre-driver models are designed not only to study the selective gene functions in the glandular and luminal epithelial compartments but also to further resolve gene functionality between two stromal cell states: the pre-decidual and decidual cell states.

While we have provided initial molecular data to support PLZF as an early mediator of P4/PGR action in the murine uterus during the peri-implantation period, genome-scale omics analysis will be essential to furnish a more comprehensive understanding of the molecular mechanisms by which PLZF exerts its physiological roles in murine uterine function. With the N-terminal BTB/POZ domain of PLZF known to be responsible for homo- and hetero-dimerization, chromatin remodeling, epigenetic transcriptional control, and formation of high-molecular-weight DNA-protein complexes [26], while PLZF’s nine C_2_H_2_ zinc finger motifs in its C-terminus are responsible for direct sequence-specific DNA binding to target genes [26], endometrial PLZF is predicted to orchestrate crucial evolutionary conserved signaling programs in response to P4. Given that we [33,35] and others [78] have shown that progestins (i.e., medroxy progesterone acetate (MPA)) induce PLZF in HESCs [33,35,78], coupled with the fact that MPA is frequently used in female reproductive medicine for the treatment of endometriosis [79], gynecological malignancies [80], postmenopausal hormone therapy [81], uterine fibroids [82], and contraceptives [83], future investigations into the molecular mechanisms that underlie this endometrial C_2_-H_2_ zinc finger transcription factor are warranted.

In summary, conclusions from these studies are that PLZF is indispensable for P4-dependent endometrial receptivity and decidualization and that PLZF may mediate these endometrial responses in a cell-type-specific manner.

## 4. Materials and Methods

### 4.1. Ethics Statement

Mice were humanely treated, and surgical procedures were conducted in accordance with the guidelines described in the Guide for the Care and Use of Laboratory Animals (“The Guide”, 8th edition, 2011), published by the National Research Council of the National Academies, Washington, DC, USA (http://www.nap.edu (accessed on 2 January 2024)). Before animal experiments were conducted, the animal protocol (AN-4203) associated with these studies was prospectively approved by the Institutional Animal Care and Use Committee at Baylor College of Medicine.

### 4.2. Mouse Models and Hormone Treatments

In a C57BL6J background, the *Plzf* conditional knockout mouse (*Plzf ^d/d^*) was generated by crossing our *Pgr-cre* knockin (*Pgr ^cre/+^*) mouse [36] with our recently generated *Plzf* floxed (*Plzf ^f/f^*) mouse in which exon 2 of the murine *Plzf* gene is floxed [37]. Mice were housed in temperature-controlled rooms (22 ± 2 °C) operating on a 12 h light/12 h dark photocycle in a vivarium facility at Baylor College of Medicine, which is accredited by AAALAC (Association for Assessment and Accreditation of Laboratory Animal Care). An irradiated Formulab Diet (LabDiet/Lab Supply, Fort Worth, TX, USA (#5008)) and fresh water were made available to mice ad libitum. For acute P4 treatment, six-week-old mice were ovariectomized and rested for two weeks before receiving an intrascapular subcutaneous (s.c.) injection of P4 (1 mg) dissolved in sesame oil. Ovariectomized mice injected with sesame oil served as vehicle controls. To elicit an artificial receptive state in the uterus, eight-week-old ovariectomized mice received an E2 and P4 hormone treatment regimen as previously described [56]. Briefly, mice were initially primed with a daily injection of E2 (100 ng) for two days before resting for two days. Following the two-day rest period, mice received a daily injection of P4 (1 mg) for three days. Following the three-day P4 treatment period, mice received a combination of P4 (1 mg) and E2 (50 ng); the E2 injection at this time mimics the natural E2 nidatory spike prior to embryo implantation. Mice were euthanized 15 h following the P4E2 injection to provide uterine tissue for histological and molecular analysis. An established protocol was used to induce an artificial decidual response in the uterus of an ovariectomized mouse [57]. Briefly, eight-week-old ovariectomized mice received three daily s.c. injections of E2 (100 ng). Following two days of rest, mice were administered three daily s.c. injections of E2 (6.7 ng) plus P4 (1 mg). Six hours following the third E2P4 injection, sesame oil (50 μL) was instilled into the lumen of the left uterine horn (stimulated (S)); the right horn did not receive oil (unstimulated (U)). After intraluminal instillation of the deciduogenic stimulus, mice received daily s.c. injections of E2P4 for 5 days and then weighed before euthanasia. Trimmed of mesometrial membrane and vasculature, dissected stimulated and unstimulated uterine horns from each mouse were weighed for wet-weight measurements before further analysis.

### 4.3. Fertility Analyses

To time-specific days of gestation, *Plzf ^f/f^* and *Plzf ^d/d^* females (8–10 weeks old) were housed overnight with fertility-proven wild-type male mice. The following morning, coitus was visually confirmed in the female by the retention of a postcoital vaginal plug; the morning of detecting the vaginal plug was assigned as the morning of gestation day 1 (GD1). Prior to euthanasia on specific GDs for the described studies below, pregnant mice were individually housed. For breeding trials, sexually mature *Plzf ^f/f^* control and *Plzf ^d/d^* mutant females were housed with fertility-proven C57BL/6 males. Over at least a 6-month breeding period, the date of pup delivery, the number of litters, and the number of pups per litter were recorded for each female. To elicit superovulation, 21-day-old female mice were intraperitoneally (i.p.) injected with pregnant mare serum gonadotropin (PMSG; Sigma-Aldrich, St. Louis, MO, USA (5 international units (IU)/100 μL of sterile 0.9% saline)). Forty-eight hours post-injection, mice received an i.p. injection of human chorionic gonadotropin (hCG; Sigma-Aldrich (5 IU/100 μL of sterile 0.9% saline)). Sixteen hours later, oocytes were harvested from the lumen of the fallopian tubes and counted using a dissecting microscope as previously detailed [84]. Using fallopian tube tissue that was dissected from *Plzf ^f/f^* and *Plzf ^d/d^* females at GD 2, two-cell stage embryos were retrieved by gently flushing the fallopian tube lumen with 100 μL of sterile phosphate-buffered saline (PBS). To visualize emerging implantation sites along both uterine horns of mice at GD5, a Chicago sky blue dye solution (1% in PBS; 100 μL per mouse) was injected into one lateral tail vein before mice were euthanized 2–5 min later [84]; stained reproductive tract tissues were removed first for the purposes of imaging and subsequent cellular and molecular analyses.

### 4.4. Histology and Immunohistochemical Detection

Following overnight fixation in 4% paraformaldehyde, tissues were stepwise dehydrated by sequential incubation in increased concentrations of ethanol; dehydrated and fixed tissues were cleared with xylene before paraffin embedding. Paraffin-embedded tissues were sectioned to 5 μm thickness onto slides before tissue sections were stained with hematoxylin and eosin (H&E) for general histological analysis [84,85]. Immunohistochemical visualization of PLZF protein was achieved using an anti-PLZF primary mouse monoclonal antibody (D-9 [86]; Santa Cruz Biotechnology Inc., Dallas, TX, USA, #sc-28319, 1:150 dilution) in combination with the Mouse on Mouse (M.O.M.) Elite Immunodetection kit, Peroxidase (Vector Laboratories Inc., Burlingame, CA, PK-2200). Using the BrdU in situ detection kit (BD Biosciences, San Jose CA, USA, #551321), cells in the S-phase of the cell cycle were visualized by immunohistochemical detection of 5-bromo-2′-deoxyuridine (BrdU). After immunostaining, tissue sections were counterstained with hematoxylin prior to the application of Permount solution to aid the placement of coverslips. For cell counting in general, immunopositive cells were counted within a field of 300 cells to obtain the mean number of immunopositive cells per 300 cells counted. For the majority of studies, at least 3–4 uterine fields of 300 cells were counted per mouse. Unless otherwise specified, the mean number of immunopositive cells was calculated from at least 3 mice per genotype and treatment group. Recent versions of the Photoshop and Illustrator programs within the Adobe Creative Suite software package (Adobe Systems Inc. San Jose, CA, USA (https://www.adobe.com/creativecloud/plans.html)) were used for raw image processing, compiling of image composites, and the annotation of the final figures for manuscript preparation.

### 4.5. Molecular Analysis

Experimental conditions for murine PLZF immunoblot analyses have been reported previously [33,37]. Briefly, protein concentration was obtained using the Bradford reagent (ThermoFisher Scientific Inc., Waltham, MA, USA, #23225) before protein extracts (20 μg/lane) were resolved on a 4–15% polyacrylamide-SDS gel. Resolved proteins were transferred to a polyvinylidene difluoride (PVDF) membrane. After blocking PVDF membranes with 5% nonfat milk in Tris-buffered saline containing 0.1% Tween 20 (TBST), membranes were incubated with an anti-PLZF primary mouse monoclonal antibody (D-9; Santa Cruz Biotechnology Inc. # sc-28319) or a mouse monoclonal anti-β-actin (AC-74, Sigma-Aldrich; #A2228) overnight at 4 °C. Following the primary antibody incubation step, the immunoblots were washed and then incubated with an anti-mouse IgG_1_k horseradish peroxidase (HRP)-conjugated secondary antibody (m-IgG Fc BP-HRP; Santa Cruz Biotechnology Inc., sc-525409) for 1 h at room temperature. Resultant chemiluminescence signals were detected using the SuperSignal West Pico PLUS Chemiluminescent substrate kit (ThermoFisher Scientific Inc., #34580). For quantitative real-time PCR (qRT-PCR) analyses, total RNA was prepared from uterine tissue using the RNeasy Plus Mini kit (Qiagen Inc., Germantown, MD #74134). The NanoDrop 2000 UV/Visual spectrophotometer (ThermoFisher Scientific Inc.) was used to quantitate RNA before the reverse transcription step using the High-Capacity cDNA Reverse Transcription kit (ThermoFisher Scientific Inc. #4368814). Following reverse transcription, amplified cDNA was diluted to 10 ng/μL before qRT-PCR was conducted using the Fast TaqMan 2X Mastermix (Applied Biosystems/Life Technologies, Grand Island, NY, USA, #4352042); the TaqMan assays used in these experiments are listed in Table S1. All qRT-PCR experiments were performed on the 7500 Fast Real-Time PCR system (Applied Biosystems/Life Technologies); the delta-delta cycle threshold was used to normalize expression to the internal 18S reference.

### 4.6. Serum Hormone Measurements

Using microtainer tubes containing a serum separator microguard (Becton, Dickinson and Company, Franklin Lakes, NJ, USA (#365967)), whole blood was collected from virgin and pregnant mice at GD5 as previously described [85]. To ensure complete blood coagulation, drawn blood was held at room temperature for 30 min before serum samples were separated by centrifugation at 2000 rpm for 10 min at 4 °C; serum was stored at −80 °C until analysis. Serum P4 and E2 levels were measured by the Ligand Assay and Analysis Core of the Center for Research in Reproduction at the University of Virginia (Charlottesville, VA, USA); assay details are available at https://med.virginia.edu/research-in-reproduction/ligand-assay-analysis-core/assay-methods/ (accessed on 15 October 2023).

### 4.7. Statistical Analyses

The two-tailed unpaired Student t-test (Welch-corrected) was used to estimate the statistical significance of differences between the two groups. Unless otherwise stated, data were graphically displayed as the mean ± standard error of the mean (s.e.m.). Differences between means with a *p*-value < 0.05 were considered statistically significant, with the number of asterisks indicating the level of significance: * *p* < 0.05; ** *p* < 0.01; and *** *p* < 0.001. Version 9 of the Prism software package from GraphPad Software Inc. (San Diego, CA, USA) was used for the statistical analyses in these studies.

## Figures and Tables

**Table 1:** Litter and pup number derived from *Plzf ^f/f^* and *Plzf ^d/d^* female mice after a six-month breeding period.

Genotype	N	Total Litter Number	Total Pup Number	Average Litter Number/Dam	Average Pup Number/Dam
*Plzf ^f/f^*	13	58	458	4.4	35.0
*Plzf ^d/d^*	14	0	0	0	0

## Data Availability

The data supporting the findings of this study are available within the article and Supplementary Materials.

## Supplementary Materials

The following supporting information can be downloaded at https://www.mdpi.com/article/10.3390/ijms25063451/s1.
