# Supplementary material for: Establishment of Murine Pregnancy Requires the Promyelocytic Leukemia Zinc Finger Transcription Factor
Source: Int J Mol Sci. 2024 Mar 19;25(6):3451. doi: 10.3390/ijms25063451 (PMC10970820; doi:10.3390/ijms25063451)
Supplement: Supplementary file 1 [file ijms-25-03451-s001.zip › ijms-2882672-supplementary.pdf]

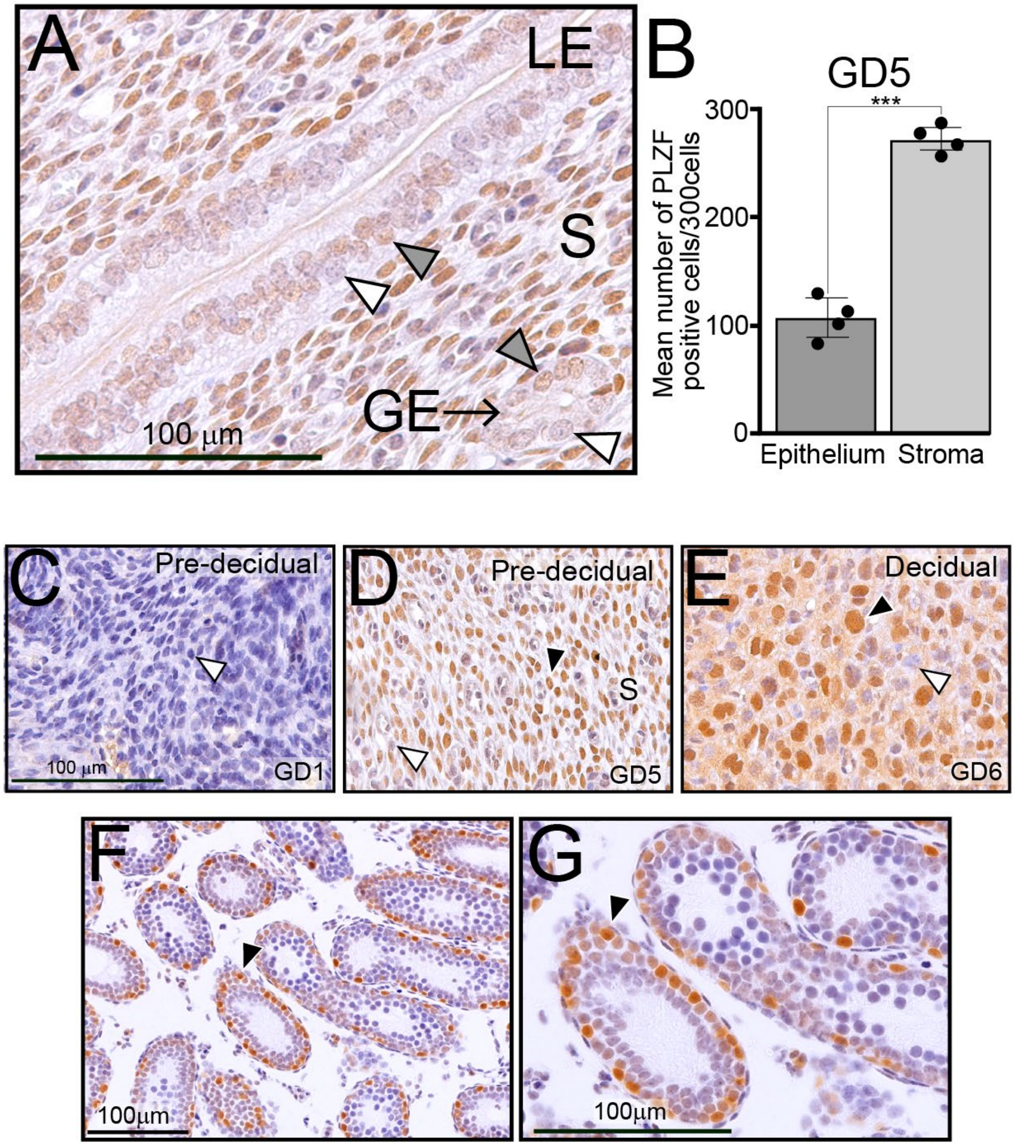

SUPPLEMENTARY FIGURE 1

Figure S1. The endometrial cellular compartments of the *Plzf* $^{f/f}$ mouse express PLZF at GD5. (**A**) Approximately 30% of the luminal epithelium (LE) expresses moderate levels of PLZF (grey arrowhead); white arrowhead indicates a LE cell scoring negative for PLZF immunopositivity. The glandular epithelium (GE) displays an equal distribution pattern for PLZF positive and negative cells (white and grey arrowheads respectively). The majority of fibroblasts in the stromal compartment are strongly immunopositive for PLZF expression. (**B**) The histogram displays the mean number of PLZF positive cells per 300 cells counted per field in the epithelium and stroma of the *Plzf* $^{f/f}$ endometrium at GD5. (**C**) Pre-decidual stromal fibroblasts are negative for PLZF expression at GD1 (white arrowhead). (**D**) Pre-decidual stromal fibroblasts are positive for PLZF expression at GD5. (**E**) Differentiated stromal decidual cells are strongly immunopositive for PLZF in the *Plzf* $^{f/f}$ decidua at GD6. As a positive control for the PLZF antibody, panels (**F**) and (**G**) show low and high power magnifications respectively of a transverse

section of seminiferous tubules within the testis from a two week old *Plzf* $^{f/f}$ mouse. Note the expected restricted expression of PLZF to the spermatogonial cell type [28].

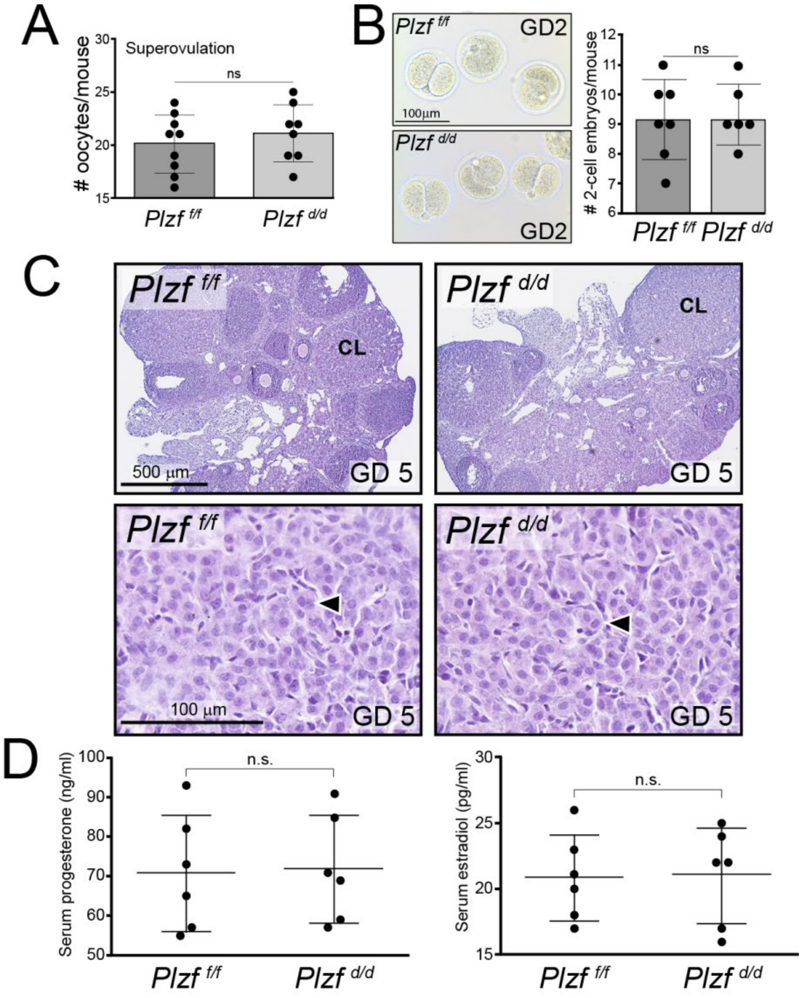

SUPPLEMENTARY FIGURE 2

Figure S2. The *Plzf* $^{d/d}$ mouse exhibits normal ovarian function. (**A**) In response to a standard superovulation hormone regimen, *Plzf* $^{d/d}$ mice produce an equivalent number of oocytes as compared with similarly treated *Plzf* $^{f/f}$ control mice. (**B**) At GD2, *Plzf* $^{f/f}$ and *Plzf* $^{d/d}$ mice produce a similar number of two-cell stage embryos. (**C**) Histological analysis of transverse sections of *Plzf* $^{f/f}$ and *Plzf* $^{d/d}$ ovaries show that both genotypes exhibit corpora lutea (CLs (top panels)) at GD5; the luteal cell morphology is indistinguishable between the two genotypes (bottom panels (black arrowheads)). Scale bars in the top and bottom left panels also apply to the right top and bottom panels respectively. (**D**) There is no significant difference in the levels of serum P4 and E2 in *Plzf* $^{f/f}$ and *Plzf* $^{d/d}$ mice at GD 5. Note a separate cohort of six mice per genotype was used to measure P4 and E2 levels.

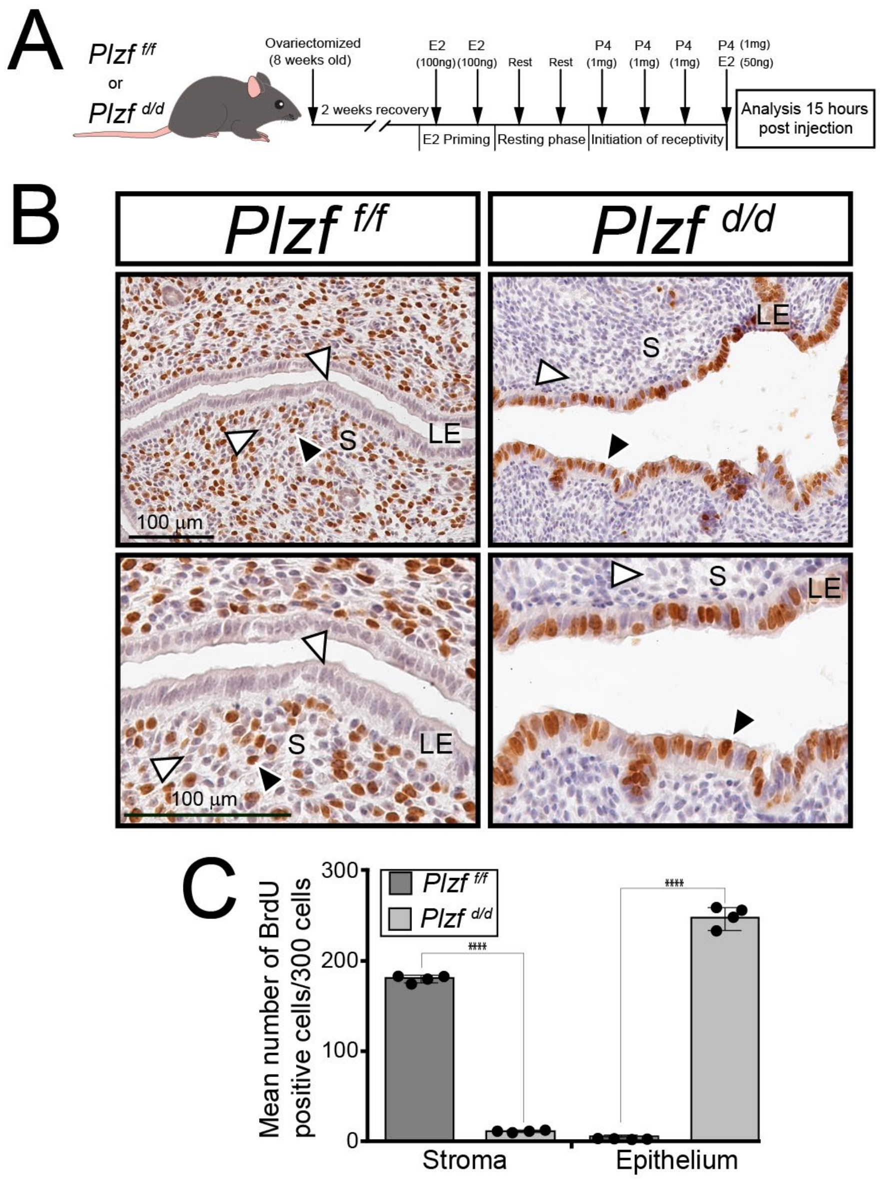

SUPPLEMENTARY FIGURE 3

Figure S3. The *Plzf* $^{d/d}$ uterus fails to attain an artificial receptive state in response to a standard estradiol and progesterone hormone treatment regimen. (**A**) The schematic outlines the standard E2 and P4 treatment to elicit an artificial receptive state in the ovariectomized mouse [40]. (**B**) Following the hormone treatment schedule described in panel (**A**), the left top and bottom panels show that BrdU staining is absent in the luminal epithelium ((LE) white arrowhead) of the *Plzf* $^{f/f}$ uterus. Conversely, the *Plzf* $^{f/f}$ stromal (S) compartment displays a significant number of cells that are positive for BrdU incorporation (black arrowhead), which are intermingled with stromal cells that are negative for BrdU incorporation (white arrowhead). Shown in the right top and bottom panels, the majority of cells scoring BrdU positivity in the *Plzf* $^{d/d}$ uterus in response to the same hormone treatment are only present in the luminal epithelium (LE). Scale bars in the left top and bottom panels also apply to the right top and bottom panels respectively. (**C**) The histogram displays the mean number of cells that are

immunopositive for BrdU in the stroma and epithelium per 300 cells counted in the *Plzf* $^{f/f}$ and *Plzf* $^{d/d}$ uterus.

Table S1. TaqMan assays for quantitative real-time PCR

Gene Name	Gene ID	Catalog number
Amphiregulin	*Areg*	Mm01354339_m1
Bone morphogenetic protein 2	*Bmp2*	Mm01340178_m1
Cytochrome P450 26A1	*Cyp26a1*	Mm00514486_m1
Early growth response 1	*Egr1*	Mm00656724_m1
ERBB receptor feedback inhibitor 1	*Errfi1*	Mm00505292_m1
Estrogen receptor 1	*Esr1*	Mm00433149_m1
Heart and neural crest derivatives expressed 2	*Hand2*	Mm00439247_m1
Histidine decarboxylase	*Hdc*	Mm00456104_m1
Indian hedgehog	*Ihh*	Mm00439613_m1
Interleukin-13 receptor subunit alpha-2	*Il13ra2*	Mm00515166_m1
Lactoferrin	*Ltf*	Mm00434787_m1
Progesterone receptor	*Pgr*	Mm00435628_m1
Prolactin family 3, subfamily c, member 1	*Prl3c1*	Mm00479148_m1
Prolactin family 8, subfamily a, member 2	*Prl8a2*	Mm01135453_m1
Promyelocytic leukemia zinc finger	*Zbtb16*	Mm01176868_m1
Transcription factor 23	*Tcf 23*	Mm00453545_m1
Eukaryotic 18S ribosomal (r) RNA endogenous control ThermoFisher Scientific	*18S rRNA*	4319413E
